# ‘Fight TB with BCG’: Mass Vaccination Campaigns in the British Caribbean, 1951–6

**DOI:** 10.1017/mdh.2014.49

**Published:** 2014-10

**Authors:** Henrice Altink

**Affiliations:** Department of History, University of York, YO10 5DD, York, UK

**Keywords:** Tuberculosis, Vaccination, Caribbean, Colonial Office, UNICEF, World Health Organization

## Abstract

Based on a wide range of primary materials, including WHO reports and Colonial Office correspondence, this article examines the UNICEF/WHO-funded mass BCG campaigns that were carried out in seven Caribbean colonies between 1951 and 1956. It explores the reasons behind them, their nature and aftermath and also compares them to those in other non-European countries and discusses them within a context of decolonisation. In doing so, it not only adds to the scholarship on TB in non-European contexts, which had tended to focus on Africa and Asia, but also to the relatively new field of Caribbean medical history and the rapidly expanding body of work on international health, which has paid scant attention to the Anglophone Caribbean and the pre-independence period.

## 1. Introduction

Mass BCG vaccination campaigns to protect people against tuberculosis (TB) were first undertaken as an emergency measure in several war-torn European countries by the International Tuberculosis Campaign (ITC), a joint initiative of three Scandinavian organisations. In March 1948, the United Nations International Children’s Emergency Fund (UNICEF) joined the ITC and not long thereafter the World Health Organization (WHO) provided it with technical expertise. In 1948, WHO had made TB control one of its priority programmes, triggered by the fact that TB was one of the leading causes of death in all of its member states and there was increasing evidence that BCG and anti-TB drugs were effective. As a result, the ITC was able to extend its work to other European countries and also to Asia, the Middle East, North Africa, and Latin America. In most countries the ITC undertook mass vaccination campaigns, consisting of mobile teams, who tested the eligible population with tuberculin and vaccinated negative reactors with liquid BCG. But in a few, such as India and Mexico, it sent out demonstration teams that taught local staff how to undertake a mass vaccination campaign. When the ITC handed over to UNICEF and WHO in 1951, it had conducted campaigns in 23 countries, testing 30 million and vaccinating some 14 million people.^1^

While the formation of the ITC and its campaigns in Europe and India have received scholarly attention,^2^ the mass BCG campaigns in non-European developing countries carried out by UNICEF and WHO after their take-over of the ITC have thus far been largely ignored.^3^ By examining BCG campaigns in seven British Caribbean colonies in the 1950s – Barbados (1956), Grenada (1954), Guyana (1954–5), Honduras (now Belize, 1953–4), Jamaica (1951–3), St Kitts (1953–4) and Trinidad (1952–4) – a significantly more successful area of BCG vaccination than India where significant resistance occurred,^4^ this study will enhance our understanding of the non-European BCG campaigns, which constituted WHO’s largest field programme in the 1950s. Focusing mainly on Jamaica, which was the first Caribbean colony to undertake a BCG campaign, it will examine the reasons behind, the nature and aftermath of the Caribbean campaigns, compare them to those in other non-European countries and discuss them within a context of decolonisation.

To meet nationalist aspirations and pre-empt US and UN pressure to dismantle the Empire, post-war British governments were committed to a policy of colonial political advancement. By 1956 when the BCG campaigns came to an end in the Caribbean, the political process of decolonisation in the region had significantly progressed. All colonies had adopted universal suffrage, while Jamaica, Barbados, Trinidad and several others also had responsible government in internal matters and plans for a West Indies Federation, through which independence for the region was to be achieved, were in an advanced state. Decolonisation, however, was not just a political process. A sound economic foundation on which to build social and political structures was seen as an essential prerequisite for granting colonies self-government.^5^ To that purpose, in 1940, the Colonial Development and Welfare Act (CDWA) was passed, which allocated £50 million to the colonies for development projects for the next ten years. Five years later the sum was increased to £120 million and the period extended to 1956. The CDWA, which emphasised social over economic development, arose out of a mixture of humanitarian concern – the need to alleviate the most direct effects of colonial poverty – and a desire to pre-empt criticism abroad about the way Great Britain managed its empire.^6^ While some scholars have claimed that the CDWA was the ‘first step in a positive and constructive policy which led on to the post-war policy of “political advancement”’,^7^ most agree that the CDWA ultimately served more to reinvigorate than end empire.^8^

By discussing the Caribbean campaigns in a context of decolonisation and comparing them to those in other non-European countries, this study adds to three overlapping sets of scholarship. First, it contributes to existing work on TB. Since the publication of Randall Packard’s *White Plague, Black Labour: Tuberculosis and the Political Economy of Health and Disease in South Africa* (Berkeley, CA: University of California Press, 1989), various studies have been published on TB outside Europe and North America, including several on the (former) British Empire. Most of the latter are more concerned with epidemiological and pathological understandings of TB and institutions to cure the disease in the colonies than with attempts by public health authorities to control and prevent it, and they also focus mainly on India and Africa and to a lesser extent the white dominions.^9^

Secondly, this study augments the scholarship on Caribbean health and medicine. While colonial medical history has been an established sub-discipline within the history of medicine since the 1980s, Caribbean medical history is a relatively new field.^10^ It has focused mainly on the development of public health in the decades preceding the Second World War, development that was aided by the work of the Rockefeller’s International Health Board and the 1929 Colonial Development Act (CDA), the precursor of the CDWA and the first act to make funds available from the British exchequer for colonial development. This study, in contrast, will extend the history of public health in the British Caribbean, both by focusing on the 1950s, setting out changes and continuities in the provision of public health care,^11^ and by concentrating on TB, a disease only mentioned in passing in existing studies and then in relation to one particular colony rather than, as in this study, in relation to nearly the whole of the British Caribbean.^12^

Finally, this examination of the Caribbean BCG campaigns adds to existing scholarship on international health organisations, which has shown that WHO and others stepped into the vacuum left by colonial powers after independence but has been less concerned with the work carried out by these organisations in the pre-independence era, especially at a time when their role was still evolving as was the case at the start of the BCG campaigns in the Caribbean.^13^ The campaigns were, as mentioned, not the first international health initiatives in the British Caribbean and took place alongside other UNICEF and WHO health campaigns and preceded many large-scale initiatives by the Pan American Health Organization (PAHO). Yet apart from the Rockefeller hookworm campaigns and other Rockefeller health work in the region, historians working on international health organisations have largely ignored the British Caribbean.^14^

As the Caribbean colonies became more independent in the 1950s, fewer demands were placed upon them to collect statistical and other data about the state of the colony and forward this to the Colonial Office, including annual medical reports. After independence, which started with Jamaica and Trinidad in 1962, the process of forming or reorganising Ministries of Health affected the collection of health statistics. As a result, this study cannot assess whether the BCG campaigns succeeded in reducing TB.^15^ Yet existing annual medical reports and other material ranging from Colonial Office correspondence and articles in Caribbean medical journals to WHO reports of the BCG campaigns allow it to set out not just how and why the campaigns came about in the Caribbean and how they compared to other non-European campaigns but also whether the Caribbean colonies succeeded in making BCG an integral part of their TB work after the campaigns ended. But before exploring the beginning, nature and aftermath of the Caribbean BCG campaigns, a brief overview of TB in the region up to the early 1950s is appropriate.

## 2. The Problem of Tuberculosis

As compulsory notification of TB was not adopted in most Caribbean colonies until the early 1910s, information about the incidence of the disease prior to this date is scarce. Based on a few existing statistics and some general literature, Dr Santon Gilmour, who carried out a TB survey in several Caribbean colonies in 1943–4 funded by the National Association for the Prevention of Tuberculosis (NAPT) and the Colonial Development and Welfare Fund set up under the CDWA, surmised that there was little TB in the British Caribbean in the early nineteenth century and that it affected mainly the European minority. Because slaves did not interact much with Europeans as they were confined to the plantations, Gilmour argued that they must have been relatively free of the disease. After emancipation in 1838, ex-slaves moved away from the plantations and the incidence of TB in the region rapidly increased. Gilmour estimated a death rate of 700 per 100 000 of the population by the middle of the nineteenth century. By the end of the century, the death rate was down by a third, largely because people developed acquired immunity to TB. And it declined further thereafter due to improved sanitation. Yet on the eve of the BCG campaigns, as table 1 illustrates, TB was still a major problem.^16^

**Table 1:** TB 1950. Source: Pan-American Sanitation Bureau, Summary of Reports on the Health Conditions in the Americas 1950–3 (Washington, D.C.: PASB, 1954), 74–5.

Colony	Reported cases per 100 000	Deaths per 100 000
Barbados	37.3	40.7
Grenada	36.4	36.4
Guyana	67.0	50.5
Honduras	137.9	56.4
Jamaica	71.0	79.1
St Kitts	88.2	107.1
Trinidad	65.0	74.3

Because of the small quantity of milk consumed, TB in the Caribbean in the decades leading up to the mass campaigns was mostly pulmonary TB. It was largely an urban phenomenon, affecting mostly young adults, and usually taking a rapid and fatal course. The white minority,^17^ which was the most affluent group in society, had the lowest incidence and death rates, followed by the Indo-Caribbean population, which in Trinidad and Guyana constituted about a third of the population, while the black majority suffered the highest rates as they tended to be located more in overcrowded urban areas.^18^

While in most Caribbean colonies the TB death rate declined after the Second World War, the disease was still a ‘serious problem’ on the eve of the campaigns. For example, in St Kitts in 1950 the TB death rate was nearly five times that of England and Wales and the disease was the main cause of death.^19^ But what led the Colonial Office in particular to define TB as a ‘serious problem’ in the Caribbean was the region’s rapidly growing population, whose poor wages and living conditions did much to increase susceptibility to the disease. Between 1940 and 1950, the population increased from 2 692 400 to 3 243 300.^20^ Slum clearance, the building of new houses and the creation of new jobs failed to keep step with this population growth. Hence many people not only lived in heavily overcrowded ‘yards’ with poor sanitation but also suffered from unemployment, underemployment and low wages, which does much to explain the pockets of undernutrition or malnutrition that contributed to the high incidence of the disease.^21^

That TB incidence and death rates varied across the region, as illustrated in table 1, was because of differences in the degree of urbanisation, the development of transportation and facilities to control the disease. Urbanisation and with it the development of slums occurred first in the larger and more prosperous colonies. For example, by 1943 some 85% of Jamaicans already lived in the capital Kingston, which had a population of 201900.^22^ In the smaller and less prosperous colonies, such as St Kitts, it was not until the Second World War that transportation improved and young people from the country moved in large numbers to urban areas in search of work. Because of their low wages, they tended to live in the most infectious areas and when they contracted TB, they usually moved back home and thereby helped to spread the disease into rural areas.^23^

The larger and more prosperous colonies were also the first to adopt measures to control TB, ranging from isolation wards in poorhouses and TB clinics to sanatoria. It was first voluntary organisations and later the (central and local) government, and in the case of Jamaica also the Rockefeller Foundation, that undertook these initiatives. For instance, the first TB clinic in the region, which predated many others in the British Empire, was set up in Guyana in 1908 by the Society for the Prevention of Tuberculosis.^24^ A similar organisation had been established in Trinidad two years earlier but it only opened a clinic in 1917. It was the Rockefeller Foundation that established the first TB clinic in Jamaica in 1928, the same year that the island’s Anti-Tuberculosis League was formed. On the eve of the BCG campaigns, there were permanent TB clinics in Guyana, Trinidad, Jamaica and Honduras, where people could get tested, and receive food and medication, and which also employed nurses that undertook case-finding and follow-up work, while most health centres in the smaller colonies held weekly TB clinics.^25^

In the early 1930s, Guyana, Trinidad, and Jamaica started to isolate TB sufferers in poorhouses and government hospitals and set up special TB hospitals for advanced cases. It was not until the 1940s that smaller colonies erected TB isolation wards in their poorhouses and hospitals. Just after the Second World War broke out, large sanatoria, made possible by the CDA, were opened in Jamaica and Guyana.^26^ Trinidad had also requested CDA funds for a sanatorium but its application had been turned down because the colony was seen as capable of paying for the institution itself.^27^ The island was more successful under the CDWA. This act had been largely facilitated by the West India Royal Commission (WIRC), which had investigated the reasons behind labour riots that had taken place in the Caribbean between 1934 and 1938.^28^ In its preliminary report released in 1940, the WIRC recommended not only constitutional changes, such as an extension of the franchise and increase in the power of elected members of the Legislative Councils,^29^ but also various social welfare measures and a financial scheme to fund them.^30^ With CDWA funding, a large sanatorium was built in Trinidad, which opened its doors in 1950, followed by smaller sanatoria in Grenada and Honduras. The treatment offered in these institutions included ‘rest cure’, surgery for ‘hopeful cases’, and on the eve of the mass campaigns also the use of the drugs streptomycin, PAS, and INAH on a small scale.^31^

In the immediate post-war period, the governments in Jamaica, Trinidad and Guyana took full responsibility for TB work by establishing a separate TB division within their medical department led by a TB officer. They also sent medical staff abroad for training in TB work.^32^ In the smaller colonies, TB control work was less co-ordinated and carried out mostly by non-specialists, including District Medical Officers (DMOs – general doctors in a particular district), Medical Officers of Health (MOHs – public health doctors in a particular district) and public health nurses, and in non-specialist institutions, such as general hospitals and rural health centres.^33^

There was thus an increase in efforts to control TB in the Caribbean in the years leading up to and following the Second World War, which exceeded that of many other British colonies and was largely made possible by the CDA and CDWA.^34^ Yet there was variation between the colonies, with on the one hand the big three – Jamaica, Trinidad and Guyana – which offered almost as many beds as the annual number of TB deaths and had specialist staff, institutions and equipment, and the smaller colonies on the other, where TB work was integrated into the general health services. St Kitts, for example, only had twenty-three beds in a general hospital, a weekly TB clinic held at a health centre and several public health nurses that did case-finding and follow-up work.^35^ It was largely because of their financial capabilities that the smaller colonies had less advanced TB schemes. All colonies could apply under the CDWA for grants or loans to set up specialist TB institutions but smaller colonies were reluctant to do so because they would have to pay part of the costs and also the wages of the institution’s staff, which were not covered by CDWA funds.^36^ They did, however, receive CDWA funding, like the larger colonies, for water supply, nutrition and sanitation projects; rural health centres, hospitals, school medical services, bureaus of health education and public health nursing schemes; and house construction and land settlement schemes. Although these schemes were limited by material resources and in some places also popular resistance, they did help to control TB by tackling overcrowding in urban areas, raising awareness of TB, and making it easier for tubercular people to seek medical treatment.^37^

It was not only the fact that BCG was available for application on a mass basis thanks to the ITC that led in the late 1940s to calls by doctors from within the region for the wide-scale use of BCG but also the rate of population growth and increase in the number of young people migrating to urban areas. In May 1948, Dr Richard Cory, the doctor in charge of the Jamaican sanatorium, proposed that all schoolchildren be vaccinated. Not long thereafter, BCG was procured from the Henry Phipps Institute in Philadelphia and administered to some 400 tuberculin negative reactors, including several student teachers and probation nurses. The results of these vaccinations led the TB officer to recommend the construction of a BCG factory as well as the vaccination of all school leavers in rural parishes. The plans for a BCG factory were abandoned, however, when WHO announced it would set up such a factory in Mexico City but vaccinations continued to be administered on a small scale until the start of the mass vaccination campaign in October 1951.^38^ In Honduras and Trinidad too, the senior medical officers favoured the wide-scale use of BCG and in the latter colony the vaccine was administered to probation nurses and some others before the start of the campaign, while St Kitts had already started to vaccinate schoolchildren by the time it sought UNICEF/WHO support.^39^ These small-scale BCG programmes were not the first attempt in the region to control TB by means of vaccination. In 1939, the Rockefeller Foundation had started a controlled trial with heat-killed tubercle bacilli vaccine in Jamaica. It began with the vaccination of schoolchildren in Kingston and was gradually rolled out to others. By 1940, some 11 000 people had taken part but the trial came to an end in 1942 when it appeared that there was little difference between the vaccinated and non-vaccinated groups.^40^

Thus like many of the other non-European countries where UNICEF/WHO carried out BCG campaigns, the British Caribbean was an ideal location. First of all, TB here was a serious problem because of the rapidly growing and highly mobile population. Second, most of the Caribbean colonies were small, both in terms of land mass and population, and had a decent transportation infrastructure. Third, the TB services as well as the general medical services were relatively developed largely because of the CDA and CDWA so that by the early 1950s even the smaller and less densely populated colonies had several general hospitals, rural health centres, a district nursing system, child welfare centres and a school medical service.^41^ Fourth, as a result of several decades of TB work, the Caribbean population was well aware of the dangers of TB. And finally, the senior medical officers in the region, key to any mass campaign, regarded BCG as an important means to control TB. But because the British Caribbean was not independent, the colonial governments in the region had to seek approval from the Colonial Office for a UNICEF/WHO-sponsored campaign. The following section explores the Colonial Office’s gradual acceptance of BCG as a means of addressing the problem of TB in the colonies and mentions some other factors that facilitated the Caribbean BCG campaigns.

## 3. Negotiating a Campaign

From 1949 onwards the Colonial Office began to address TB seriously. In addition to ordering more TB surveys, it appointed Professor Heaf from the University of Cardiff as TB consultant. Heaf scrutinised all TB-related requests for CDWA funding and more generally helped the Colonial Office to develop a ‘co-ordinated attack on the disease’.^42^ That BCG soon became an integral element of this ‘attack’ was first of all because large-scale evidence from trials had become available showing its efficacy. In the years leading up to the Second World War, France and the Scandinavian countries had begun to administer BCG on select groups. Results of these trials and of smaller ones carried out in North America led several leading TB experts at the 1947 Commonwealth and Empire Tuberculosis conference, sponsored by NAPT and supported by the Secretary of State for Commonwealth Relations, to conclude that the ‘safety’ and ‘efficacy’ of BCG was ‘beyond dispute’ and that it was of particular value in the colonies, where ‘ordinary control is difficult’. Professor William Tytler from the Welsh National School of Medicine, for instance, said that ‘child vaccination may not only be of value’ in the colonies but ‘may prove to be the most effective, as it is one of the least expensive means of prevention’.^43^ And there was even more endorsement for BCG at the 1949 Commonwealth and Empire Tuberculosis conference, which included, amongst others, a paper on BCG vaccination in India that concluded that ‘it could reduce considerably the morbidity and mortality rate’.^44^ By that time, there was also less opposition to the use of BCG within the UK. In 1949, the vaccine was offered to nurses and a year later the Medical Research Council began a trial with schoolchildren.^45^

Professor Heaf was not as convinced as some of the TB experts at the two Commonwealth and Empire Tuberculosis conferences about the efficacy of BCG. For example, he stressed in meetings of the Colonial Advisory Medical Committee that BCG did not afford ‘permanent protection’ and that its usefulness was doubtful in ‘advanced age groups’. Yet he was also of the opinion that UK methods to tackle TB, such as the sanatorium regime of good food, exercise and fresh air, would not work in the colonies, where emphasis should be placed on prevention rather than treatment, and that as such BCG had a role to play. Like Dr Wilson Rae, the chief medical officer in the Colonial Office, he particularly envisioned a role for freeze-dried BCG as the live vaccine lost its potency after fourteen days and was thus not suitable for use in many colonies.^46^

That BCG became part of the ‘co-ordinated attack’ on TB in the colonies should furthermore be seen in light of the demand for it from within the colonies in the immediate post-war period. Not just Caribbean but also some African and Asian colonies were keen to vaccinate if not whole populations than at least select groups. They asked the Colonial Office to help them secure supplies of BCG and fund vaccination campaigns.^47^ And finally, WHO’s endorsement of BCG for developing nations made the Colonial Office more receptive to the use of BCG in the colonies. In September 1948, WHO asked the Colonial Office if the colonies wanted to use its TB services, which ranged from medical literature, fellowships and visiting experts to demonstration teams and BCG vaccination (through the ITC). The Colonial Office responded that it would have to consult each colony individually because of its policy to guide colonies towards responsible self-government and also the fact that a mass BCG campaign would involve considerable costs for a colonial government as any government that entered into an agreement with the ITC had to pay a share of the costs.^48^ Two years later, WHO asked the Colonial Office to consider its TB scheme for ‘countries with undeveloped or underdeveloped programmes’. BCG vaccination, which was seen as the ‘only practical way so far known for producing specific resistance against tuberculosis (even if this resistance is not absolute)’, played a central role in this programme.^49^

As a result of these various factors at home and abroad, in May 1950 the Colonial Office decided to ‘aim at B.C.G. vaccination on the widest possible scale’. It not only made arrangements with the Pasteur Institute to supply freeze-dried BCG to colonies who wanted it, such as Trinidad, but also supported colonies who applied to the ITC, including Singapore and Malaya, where BCG campaigns started in the autumn of 1950. Although never overtly articulated, support for colonies applying to the ITC was largely influenced by financial considerations as a significant part of the vaccination campaign would be carried by neither the colonial nor the imperial governments, and such a campaign was of course also infinitely cheaper than slum clearance, sanitation works and other non-medical methods of prevention. Yet the Colonial Office stressed, like WHO, that BCG did not offer absolute immunity and would only be effective if part of a comprehensive TB-control scheme.^50^

But it was not only the Colonial Office’s endorsement of the widespread use of BCG that facilitated the mass BCG campaigns the Caribbean. The establishment of a BCG laboratory in Mexico City in 1949 that allowed for the use of the liquid, and thus more effective vaccine, and the work already undertaken by UNICEF and WHO in the region – eg. a school feeding programme in Honduras, an insect control scheme in Guyana and a VD campaign in Trinidad – also played a part, as did the recruitment for mass BCG campaigns by WHO’s newly founded regional office for the Americas, which may have been largely informed by the fact that the British Caribbean was increasingly becoming part of the theatre of the Cold War.^51^ In August 1950, Dr Lourie, the recently appointed TB adviser of the Pan American Sanitation Bureau (PASB), which doubled as WHO’s regional office, visited Jamaica. He met with medical staff to ascertain the scale of TB in the island and determine whether there was a medical infrastructure for a mass vaccination campaign. Shortly thereafter, Lourie drew up a scheme based – like the ITC campaigns – on joint contributions from UNICEF and the Jamaican government that would provide for four mobile units for a period of two years starting in April 1951. The teams would test and vaccinate the population and also X-ray positive reactors.^52^

A brief comparison of how the Rockefeller Foundation came to undertake TB work in Jamaica in 1927 with how Lourie’s scheme was accepted in 1950 illustrates first of all that it was not until decolonisation that colonised people in the Caribbean themselves were able to significantly shape public health policy, and second that they fully realised the opportunities offered by the newly established UN agencies to raise health levels. In 1927, Crown Colony government was in place in Jamaica. The island had a unicameral legislature consisting of fourteen elected members and an equal number of ex-officio and nominated members presided over by a governor. The elected members did not have the right to propose monetary matters and could only overturn a monetary proposal made by the government if nine of their number voted against. Yet the governor could always overrule their veto if he deemed it a matter of ‘paramount importance’. In 1927, the Rockefeller Foundation proposed to undertake and pay for a TB survey in Jamaica. This survey used the ‘intensive method’, which was similar to that employed in the mass BCG campaigns as outlined in the next section and consisted of small teams who administered tuberculin tests to different age groups but mainly schoolchildren, starting in various neighbourhoods in Kingston and then moving on to areas outside of it.^53^ The Rockefeller Foundation liaised directly with the Director of Medical Services (DMS), Dr Wilson, who deemed such a survey of ‘considerable interest and value’ and in light of the government’s lack of funds for such an exercise, immediately tendered an official invitation to Dr Opie, a well-known American TB expert to start the survey.^54^ In 1933 Dr Opie submitted a set of proposals to combat TB based on the findings of the survey, the majority of which were implemented in following years. The elected members of the Legislative Council, however, had no real input in the implementation of Dr Opie’s proposals; they were merely asked to approve the necessary funds. For example, the government put £2 500 on the island’s budget for 1934–5 to take over the Kingston TB dispensary from the Rockefeller Foundation, which had been the headquarters of the survey. Some elected members complained about this, stating for instance that the government did not fund other parishes to tackle TB to the same degree as Kingston. But their complaints were quickly brushed aside by the governor and the sum was easily approved.^55^

Lourie had come to Jamaica as a result of enquiries made by the DMS to produce BCG locally. Yet it was not this appointed ex-pat DMS, who proposed the motion to apply to UNICEF for a BCG campaign but a locally born elected official. In 1944, Jamaica was given a new constitution that provided for a small Privy Council, dealing mainly with matters of defence; a nominated Legislative Council; an elected House of Representatives; and an Executive Council responsible for determining policy and introducing legislation. The Executive Council included five elected members, each of whom was assigned a specific area of administration for which they were spokesmen in both the House of Representatives and Executive Council. It was the elected member and quasi-Minister for Social Welfare, Donald Sangster, who moved the following resolution first in the Executive Council and later in the House of Representatives:

to approve of the [Dr Lourie’s] scheme in principle and to approve of an application being made to Colonial Development and Welfare for an island contribution of £53 098 out of a total cost of £100 848 and to the United Nations International Children’s Emergency Fund for a grant of £47 750 towards the campaign.^56^

The Executive Council fully supported the motion and was in fact ‘most anxious that this opportunity for tackling one of the island’s gravest health problems is not lost’. And on 10 October 1950, the House of Representatives equally voted in favour of Sangster’s motion.^57^

That the Jamaican BCG campaign was delayed until October 1951 was largely because decolonisation intersected with internationalism. UNICEF had been set up in 1946 as an emergency measure to provide children in war-torn Europe with food and clothing. The gradual expansion of its geographical reach and activities, including the ITC, led to questions about its future status, in particular whether it should provide long-term assistance to developing nations. The uncertainty over the organisation’s status also affected its budget. Shortly after the Jamaican House of Representatives had voted in favour of Sangster’s motion, it was told that UNICEF could only contribute $110 000 instead of the original $143 000 and that the scheme had to be revised.^58^ Because the island was a colony and not an individual member state of the United Nations, this revised application to UNICEF to pay for transport, tuberculin, BCG, medical equipment, observation visits and technical assistance had to be reviewed by both the Colonial Office and the Foreign Office. Its dependent status also meant that Jamaica could not directly negotiate the terms of the agreement with UNICEF but had to do this via the UK delegation at the United Nations in New York, which reported to the Foreign Office and this also held up the onset of the campaign.

The Secretary of State for the Colonies quickly gave his approval in principle for Jamaica’s application to UNICEF not only because of his Office’s commitment to raise colonial standards of living but also because the fund set up under the CDWA to achieve this aim was limited and there was little chance it would substantially increase in the near future.^59^ The application was subsequently sent to the UK delegation and was discussed on 1 November 1950. UNICEF, however, was only able to grant $2500 to send one doctor and two nurses from Jamaica to Ecuador to observe a BCG campaign because at the time ‘general arrangements’ for BCG campaigns after the take-over of the ITC by WHO and UNICEF had ‘not yet been submitted to the board’ and also simply because UNICEF lacked funds.^60^ The UK delegation in New York strongly advised the Jamaican government to accept this offer as it ‘left open the possibilities of further allocation later’. A month later, UNICEF’s executive approved $85 000 for all its BCG activities for 1951 and a new area allocation for Latin America of $840 000, most likely in response to the changing global political landscape – not only had North Korea invaded South Korea in June but South America had become an increasing area of concern, especially for the United States which had strategic and economic interests in the region. This was used by the UK delegation to demand that Jamaica’s BCG scheme be discussed at the next meeting.^61^ This pressure paid off as the executive board recommended at its meeting in January 1951 that $110 000 from the area allocation for Latin America be used to fund the rest of Jamaica’s BCG scheme subject to technical approval by WHO.^62^ The improvement in UNICEF’s financial position furthermore led Dr Lourie to suggest that Jamaica submit a second application to UNICEF for two mass radiography units and follow-up treatment at rural health centres of confirmed TB cases so that the scheme as a whole would offer much better diagnostic and ambulatory treatment.^63^

At the same time that the acting governor was forwarding the first Jamaican application for UNICEF assistance to the UK delegation, he also submitted an application for £55 941 under the CDWA to help Jamaica pay its share of the BCG campaign.^64^ This application was supported on the grounds that: TB was a serious problem; BCG offered the ‘best immediate prospect of tangible results’; experience elsewhere suggested that the scheme would be successful; it offered a unique opportunity for getting financial and technical support from UNICEF and WHO; and BCG would become an integral part of the island’s TB scheme because after the campaign one of the teams would be retained to vaccinate newborns and other susceptible groups.^65^

As was common for all CDWA applications from the region, Jamaica’s application was reviewed by the West Indies Development and Welfare Organisation, set up under the CDWA, which assisted local governments in drawing up submissions for the CDWA and devising development plans and by the Colonial Office. But as this application involved recurrent spending, such as the BCG team that would stay on after the campaign, it was also reviewed by the Treasury. The Comptroller in charge of the West Indies Development and Welfare Organisation and the chief medical officer and TB consultant in the Colonial Office approved the scheme because it would allow Jamaica to significantly enhance its existing TB control programme, and the Treasury was sufficiently confident that Jamaica was in a position to pay the recurrent costs.^66^

Each of the three offices had questions about the application that were passed on to relevant Jamaican officials and which delayed things considerably so that it was not until 19 June 1951 before the CDWA grant was finally approved. Further delay to the start of the campaign was caused by the Foreign Office’s decision to use the Jamaican application to draft a standard agreement between colonial governments and UNICEF setting out the obligations and rights of each that could be used for all projects involving the Fund not just the BCG campaigns, listing such general things as the immunity of UNICEF’s assets, property, income, operations and transactions from taxation. This not only illustrates that the Foreign Office expected UNICEF soon to become a permanent member of the UN (it did so in 1953) but also that it was of the opinion that the Fund had an important role to play in raising the colonial standard of living. Because Jamaica had semi-responsible government, the Foreign Office could not just discuss the formulation of an agreement for Jamaica that would become the standard for all future agreements with UNICEF, but also had to consult the Jamaican government and the Colonial Office. As a result, numerous drafts were sent between London and Kingston. On 25 July, the Foreign Office, Colonial Office, and Jamaican government had completed a final draft but then UNICEF proposed several amendments and another round of drafts started so that it was not until 2 October 1951 before the agreement was signed by the UK delegation in New York.^67^

The negotiations surrounding the first campaign in the Caribbean have been discussed here at length in order to demonstrate that in non-European, colonised territories it was not always easy for UNICEF/WHO to start a mass BCG campaign, especially when they first took over from the ITC. Even if the colonising power did not object to specialised agencies of the UN operating in its colonies – whether for financial reasons or as a useful means to stem anti-colonial propaganda –^68^ and elected local officials fully supported a campaign, it could take a long time to get it off the ground because UNICEF/WHO had to deal with various offices in both the mother country and the colony, each with their different lines of commands and priorities. And it has been suggested above that their still evolving role also made it initially difficult for UNICEF/WHO to start a campaign. Yet once a contract was signed, UNICEF/WHO were able to conduct a campaign quickly because they used the standard methods and materials pioneered by the ITC. For instance, in March 1953, the Honduras scheme was approved. Two months later staff was sent abroad for training and a mass campaign started on 13 September that lasted until 15 April 1954, covering a rugged area of some 8800 square miles and a population of 75 782.^69^ The following section will show, however, that a lack of financial and other resources made it difficult for colonies to live up to their promise to UNICEF/WHO to integrate BCG vaccination into their existing health structures.

## 4. The Campaigns and their Aftermath

While the Jamaican government, Foreign Office, Colonial Office and UNICEF were drafting a standard agreement to be used for all types of UNICEF assistance to British colonies, Trinidad submitted a request to UNICEF for a BCG campaign.^70^ Soon other colonies followed of their own accord or because, like Jamaica, they too had been approached by a WHO consultant. After a colony applied to UNICEF, a survey of local conditions was carried out by a WHO consultant and if he or she recommended a campaign, an agreement was drawn up. Because of their health infrastructure and size, the agreements varied somewhat between colonies but as in the case of the ITC campaigns they all specified that UNICEF would provide the equipment, WHO the technical expertise and local government the rest.^71^

In their Caribbean campaigns, UNICEF/WHO did not just build on but also to some extent improved the ITC campaigns by paying even more attention to local conditions. When the ITC moved outside Europe in 1949, it adapted some of its standards and procedures to make them more efficient and provide a better fit with demographic, social and cultural contexts. For instance, it began to employ lay vaccinators alongside local and international medical staff, adopted a single tuberculin test – in Europe negative reactors were tested again with a stronger dose of tuberculin –, simplified the method of record keeping, and also no longer required parental consent for schoolchildren.^72^ There was no need for UNICEF/WHO to employ lay vaccinators or international staff in the Caribbean because there were enough doctors and nurses available locally.^73^ The Jamaican medical service, for example, employed some 160 doctors and 1000 nurses in 1951.^74^ With the exception of more senior posts, such as senior medical officer of a hospital, the government medical services in the Caribbean consisted largely of locally born men and women by the early 1950s. Most of the local doctors were trained in the UK but a large number, especially those of African descent, had studied in North America. It was not until 1948 that doctors were trained within the region when a medical school was opened at the University College of the West Indies in Jamaica. Nursing schools, however, had been set up in most colonies from the turn of the century onwards and in 1943 a Public Health Training Centre was established in Jamaica that served the whole region.

As in the ITC campaigns, the doctors and nurses in the Caribbean campaigns were put into field teams. The number and make-up of the teams varied between colonies depending upon demography, geography, and financial and other resources.^75^ Jamaica, for instance, had four teams of one doctor, two nurses and a driver each but Barbados had two teams made up of only nurses as it was smaller and also had a less developed medical service. One field team from each colony was sent elsewhere to observe BCG work and upon their return trained further teams.^76^ The doctor who had taken part in observation work became the campaign organiser, while the colony’s TB officer or DMS acted as the chief of the campaign, ie. he was responsible to the three partners involved. WHO provided each colony with a TB expert for the first month of the campaign. In Jamaica and Trinidad, this was Dr Knut Osvik, WHO’s regional TB adviser, but as the campaigns progressed WHO decided that this role could be undertaken by a local doctor and appointed Dr Ronald Lampart, the Jamaican campaign organiser, as BCG consultant for the region.^77^

Lampart’s appointment and the observation work undertaken by the teams went some way towards realising the WIRC’s recommendation to unify the region’s medical services, as did the establishment of the medical school and the Public Health Training Centre, the formation of the Caribbean Council of the British Medical Association in 1951, and the conferences of heads of the medical services that were started after the War. Until the late 1930s, apart from the occasional medical conference and movement of senior staff from one colony to another, there was little interaction between the region’s medical services.^78^ The WIRC recommended unification of the medical services because it would provide local staff with ‘greater possibilities for advancement’ and ‘more opportunity for gaining wider experience’.^79^ By facilitating regional co-operation, the BCG campaigns did much to widen the experience of local staff. Only few of the campaign doctors and nurses had prior experience with TB work. Gaining this new experience allowed some to advance their career, most notably Dr Lampart, who had been a junior hospital doctor before the campaign. WHO not only made him BCG consultant but also gave him a fellowship to study for an MA in Public Health. And the two Jamaican public health nurses who had done observation work with Lampart in Ecuador managed to secure teaching positions on the basis of their BCG work.^80^

With regards to materials and techniques used, the method of record keeping and the process of field work, too, the Caribbean campaigns largely mirrored other non-European BCG campaigns.^81^ Thus also here a single tuberculin test was used; schoolchildren did not require parental consent and were re-tested to obtain information on the allergy caused by the vaccination; and the individual cards used for schoolchildren and groups cards for the general population with information about vaccine lot etc. formed the basis of monthly statistical reports that were forwarded to WHO’s Tuberculosis Research Office in Copenhagen.^82^ And like the ITC teams, the Caribbean field teams also first covered urban areas and then moved to rural districts, testing and vaccinating schoolchildren – a convenient controllable population – before the general population. Upon arrival in a new district, the teams held meetings with local health departments, teachers and local authorities to explain the campaign; selected sites for vaccination centres; and used public lectures, film shows and loud-speaker propaganda to ask people in the area to come forward for testing.^83^ Government and medical authorities supported the work of the field teams by organising publicity drives. In Honduras, for example, publicity committees were formed in all districts several months before the start of the campaign, consisting of government officials, members of the Legislative Council, and representatives of the schools, churches and voluntary organisations. All medical practitioners in the colony officially approved the BCG campaign and school managers promised that teachers would actively co-operate with the campaign, while the DMS formally opened the campaign with a broadcast on BCG that was reprinted in all papers.^84^

That UNICEF/WHO paid considerable attention to local conditions can be seen especially in the age limits adopted in the Caribbean campaigns. In Jamaica, UNICEF/WHO set out to test people aged 0–20 in urban and 0–30 in rural areas but it set other age limits for other colonies depending on the survey carried out before the start of the campaign and a colony’s resources. In St Kitts, for instance, UNICEF/WHO selected the age groups 0–25 in urban and 0–45 in rural areas.^85^ The Jamaica campaign, however, soon proved that vaccinating children under the age of one was impracticable in the field and none of the Caribbean campaigns therefore vaccinated newborns.^86^ In most colonies the upper age limit was also quickly adjusted. In both Jamaica and St Kitts, for instance, it was raised to 50 because many parents, who accompanied their children to testing centres, asked to be tested.^87^

The raising of the age limit may explain why the Caribbean colonies achieved a higher coverage rate than many other non-European countries, ranging from 40% to nearly 70% of the total population.^88^ Even with regards to the main target group – children aged 0–14 – the region compared favourably. For example, in Jamaica 52% of children were tested compared to 21% in El Salvador and 22% in Malaya.^89^ And the Caribbean colonies also stood out from other non-European countries in terms of the number of people who had their test read, which averaged 95% whereas for instance in Egypt it was only 65% .^90^ Besides the factors already mentioned in the first section, the relative success of the Caribbean BCG campaigns should also be seen in light of the use of local doctors and nurses and the high rate of school attendance – some 75% of all children in the British Caribbean aged 5 to 14 were enrolled in schools in 1949–50.^91^ This is not to say that there was absolutely no objection by the local population to the campaigns. For example, just before the campaign was to start in the Jamaican parish of Portland, *The Gleaner*, the island’s biggest-selling newspaper, published an extract from the *South African Medical Journal* that raised doubts about BCG. Although the medical department quickly issued a statement, published on the front page of the paper, that ‘there have been no reports of any ill effects suffered by anyone who has had the vaccine’, the damage was done; most Jamaican parishes had a coverage rate of 45% but in Portland it was only 29%. Yet resistance such as in Portland was the exception rather than the rule.^92^

**Table 2:** Results of tuberculin tests. Source: WHO reports.

Colony	Percentage of positive reactors
Barbados	47
Grenada	32
Guyana	39
Honduras	50
Jamaica	40
St Kitts	63
Trinidad	34

The Caribbean colonies not only differed from other non-European countries in terms of the high coverage rate and percentage of tests read but also in the number of positive reactors (see table 2), which were higher than in Europe but lower than in the Middle East and Asia, where TB services were less developed. In Greece, for instance, positive reactors made up 28.9% but in India 59%.^93^ The percentage of positive reactors varied across the region depending on such factors as the extent of urbanisation and development of TB services. Also varying between the colonies were programmes for positive reactors. Jamaica had the most comprehensive scheme. Positive reactors aged 15 to 30 were given a coupon for a free X-ray, carried out by mass radiography units that had only arrived in the island four months after the start of the campaign. Many positive reactors also had bacteriological tests done and if found tubercular, were referred to one of the specialist institutions or given ambulatory treatment.^94^ Guyana also used mobile mass radiography units, offering not just positive reactors but everyone a free X-ray. The first few months of its mass radiography campaign, however, were marked by severe mechanical problems.^95^ Honduras, on the other hand, did not obtain a mass radiography unit until the end of the campaign.^96^ And although no such units were included in the agreements between UNICEF/WHO and governments of St Kitts, Grenada and Barbados these colonies did try to offer all positive reactors an X-ray.^97^

That not all agreements between UNICEF/WHO and the colonial governments included mobile mass radiography units to test positive rectors and that in many colonies they were not in place by the start of the campaign suggest that the focus of UNICEF/WHO at the time was on the prevention rather than the cure of TB and in the case of WHO also with data for its research into tuberculin sensitivity. WHO’s reports of the Caribbean BCG campaigns, for instance, zoom in on the percentage of positive reactors, tuberculin sensitivity and the re-testing of schoolchildren but omit to state what further tests were offered to positive reactors, what proportion was eventually diagnosed as tubercular, and what treatment these patients were given.^98^

WHO’s conviction that BCG was the most practical and cost-effective method of prevention against TB can also be seen in its decision that countries could only retain the testing kits, vehicles, labs and other equipment if they submitted a plan for the continuation of BCG vaccination. While some Caribbean colonies aimed to make BCG vaccination a routine procedure in child welfare centres, the majority proposed to test and vaccinate schoolchildren. It was not just the distribution of positive reactors amongst the age groups that determined their decision but also such factors as existing medical infrastructure and degree of economic development. In Honduras, for instance, there were some eighteen, well-attended child welfare centres. The colony therefore decided that a nurse would visit each centre once a year and test and vaccinate all children aged one and a half.^99^

To what extent did the colonies live up to their promise to continue BCG vaccination? The few annual medical reports available for the late 1950s and early 1960s and other sources indicate that some colonies first undertook a follow-up BCG campaign before taking steps to test and vaccinate schoolchildren or babies. In Barbados, for instance, a follow-up campaign started in April 1957, when all schools were visited and entrants tested as well as children that had not shown a satisfactory take in the mass campaign. Thereafter, one nurse was employed full-time for BCG work, working both in the St Michael’s TB clinic and in schools.^100^ Trinidad and Guyana also appointed full-time BCG nurses. The latter colony, for instance, had a permanent BCG team made up of a health visitor and two nurses, who tested and vaccinated schools entrants and from 1961 onwards also school leavers.^101^ Jamaica, on the other hand, did not set up a permanent BCG team to vaccinate babies, as it had promised in its CDWA application, but instead opted for the testing and vaccination of schoolchildren and added this to the duties of parochial health nurses.^102^

Thus in most Caribbean colonies BCG vaccination was placed under the direct control of the central health department and was part of a larger specialist programme to control TB that also included clinics, sanatoria and so on, but in some colonies it was integrated in the general health services and was, like most other preventive work, made the responsibility of local health departments. That the Jamaican health nurses had visited only half of all schools by 1960 because they were ‘so smothered by other duties’ seems to suggest that the general approach was less successful than the specialist.^103^ However, as the permanent BCG teams were very small, they too struggled to meet their targets. The Trinidad team, for instance, had tested a total of 108 308 schoolchildren and vaccinated some 61 270 by 1960 but it never succeeded in visiting all 400 schools in the island on an annual basis.^104^

That the Caribbean colonies did not make more staff available for BCG work was largely because of their financial and human resources. For example, by the late 1950s Guyana and several other colonies began to experience a shortage of nurses as many migrated to America and Britain because of higher wages. And while CDWA money was used in some places like Jamaica to fund the BCG campaign, this source of funding not only came to an end in 1958 with the creation of the West Indies Federation but was also only intended for the development of social welfare services and not their upkeep. The BCG continuation work, then, had to be paid for from normal revenue but nearly all colonies at the time experienced a trade deficit because of an increase in the price of imports, especially colonies that lacked a principal export crop and did not have extractive industries.^105^

That the relative success of the BCG campaigns in the Caribbean was not sustained in the late 1950s and early 1960s should also be seen in light of the colonies’ long-standing bias against preventive medicine. While preventive medicine has always played second fiddle to curative medicine,^106^ this was especially the case in colonial settings. Before the Second World War, for instance, only 8% of all medical expenditure in Barbados was on preventive medicine.^107^ While this pre-war bias was largely because government medical services were set up before the development of preventive medicine and most doctors were trained in curative medicine, racial and colour biases of both the medical and political establishment also played a role. Preventive medicine in the Caribbean targeted the predominantly dark-skinned lower classes. Local politicians, whether white or black, tried as much as doctors and other members of their class to distance themselves from this group and advance the interests of their own. Hence, they were usually more in favour of the building of new hospitals than the establishment of child welfare clinics, a public health nursing system etc.^108^ The medical establishment’s bias against preventive medicine and the black middle class’s prejudices against their lower-class and invariably darker-skinned brothers and sisters was so engrained that even with the CDWA-funded expansion of preventive medicine and the gradual ‘blackening’ of local legislatures in the years surrounding the BCG campaigns, the Caribbean colonies failed to achieve an optimal balance between preventive and curative services.^109^ And this combined with the afore-mentioned economic conditions meant that TB did not feature much in budget discussions and when it did, politicians were more inclined to vote sums in favour of TB clinics, hospitals, sanatoria and ambulatory drug regimes than vaccination, case-finding and follow-up work.

## 5. Conclusion

This case study has shed light on the early stages of UNICEF/WHO’s take-over of the ITC, when BCG campaigns were extended to many non-European countries, including various colonies. It has shown that UNICEF/WHO did not change the work pioneered by the ITC but made its standard methods of testing and reporting even more efficient by adapting them as much as possible to local circumstances. Thus in the Caribbean, UNICEF/WHO relied solely on local staff, even appointing a local doctor as TB consultant for the region, and also raised the age levels largely in response to demands by adults to be tested. Yet the campaigns were about the prevention not the cure of TB; UNICEF/WHO only provided X-ray units to deal with positive reactors but not for all Caribbean colonies. The BCG campaigns did not differ in this regard from other campaigns undertaken by WHO in its first decade. It was not just limited funds but also, as Sunil Amrith amongst others has argued, a strong belief in ‘the magic bullet’ that led the organisation to prioritise prevention.^110^

That UNICEF/WHO approached first Jamaica and later also other British Caribbean colonies is not surprising as various factors were in place in the region that seemed to guarantee a successful campaign: the size of the colonies; a relatively well-developed transport and medical infrastructure; a population well aware of the dangers of TB as a result of years of campaigning by local Anti-TB societies; a high level of school attendance; and medical departments keen on administrating BCG on a large scale. And the campaigns in the region were indeed a success. Not only did they achieve a very high testing rate amongst schoolchildren but also amongst adults, and contrary to some other non-European countries like India and Mexico there was hardly any local opposition to the campaigns. They were also a success for some of the locals employed in the campaigns as they acquired expertise that allowed them to further their careers. And even the Imperial government must have regarded them as a success. Not only did the campaigns allow it to save money in tackling one of the most deadly diseases in the Caribbean at a time when the treasury showed little willingness to increase the CDWA fund but they also facilitated closer co-operation between the medical services in the region as staff from one colony did field work in another, which was seen as an important pre-requisite for independence that was to be achieved through a West Indies Federation.

But the success of the campaigns was not sustained in the years following, when most colonies struggled to make BCG an integral part of their TB work as a result of a lack of funds and in some places also medical staff. This failure, however, should also be seen in light of the fact that while the process of political decolonisation was speeded up in the years following the BCG campaigns, even leading in some colonies to a proper ministerial system, many social and political structures remained unchanged, including the medical services. Thus in Jamaica, for instance, the Department of Health that used to be led by the DMS was renamed the Ministry of Health in 1953 and a member of the leading party – Rose Leon – was appointed as the first Minister of Health. Yet the Ministry like its predecessor remained divided between a preventive/public health unit and a curative/clinical unit. In addition, there continued to exist in Jamaica and elsewhere a division between centrally funded/organised health services and locally funded/organised health services that did most of the preventive health work. These dual divisions, then, made it far from easy to make BCG an integral part work of TB work as did the fact that the main focus of the medical services in terms of money, staffing and administrative support remained firmly on curative work.

The process of decolonisation, however, was not only the backdrop to the aftermath of the BCG campaigns. The medical infrastructure which made the region such an ideal place for UNICEF/WHO to carry out mass BCG campaigns was significantly developed in the immediate post-war period with funding made available under the CDWA, an act based on the assumption that a sound economic foundation on which to build social and political structures was an essential prerequisite for granting self-government. And constitutional reforms in the 1940s allowed elected members of the legislatures in the region for the first time to significantly shape health policy. Many elected officials realised that both their colony’s revenue and CDWA funds, which were restricted to the starting-up costs of social welfare services, were insufficient to raise health levels of the population and were therefore keen to use the opportunities offered by the newly formed agencies of the UN. Yet increasing independence in the region also affected the campaigns in a more negative way in that it delayed their onset as UNICEF/WHO had to negotiate with different offices in both the mother country and the colonies at a time when their own role was still evolving, which also affected the the start of the campaigns. But in spite of these problems, the mass BCG campaigns in the British Caribbean were a success and alongside the increasing use of anti-TB drugs played an important role in bringing down the incidence and death rates of TB in the region.

## Footnotes

1. *International Work in Tuberculosis, 1949–1964*(Geneva: WHO, 1965), 10–13; George W. Comstock, ‘The International Tuberculosis Campaign: A Pioneering Venture in Mass Vaccination and Research’, *Clinical Infectious Diseases*, 19, 3 (1994), 534–6.10.1093/clinids/19.3.5287811874

2. On the formation of the ITC and a brief overview of its work, see Comstock, *op. cit.*(note 1); John Farley, *Brock Chisholm, The World Health Organization and the Cold War* (Vancouver: UBC Press, 2008), ch. 8. On the ITC campaign in India, see Niels Brimnes, ‘Vikings against Tuberculosis: The International Tuberculosis Campaign in India, 1948–1951’, *Bulletin of the History of Medicine*, 81, 2 (2007), 407–30; Niels Brimnes, ‘BCG Vaccination and WHO’s Global Strategy for Tuberculosis Control 1948–1983’, *Social Science and Medicine*, 67, 5 (2008), 863–73; Christian W. McMillen and Niels Brimnes, ‘Medical Modernisation and Medical Nationalism: Resistance to Mass Tuberculosis Vaccination in Post-Colonial India, 1948–1955’, *Comparative Studies in Society and History*, 52, 1 (2010), 180–209.

3. If the post–1951 campaigns are addressed, it is usually as part of a larger discussion about WHO’s vaccination campaigns.

4. On resistance in India, see Niels Brimnes, ‘Another Vaccine, Another Story: BCG Vaccination against Tuberculosis in India, 1948 to 1960’, *Cien Saude Colet*, 16, 2 (2011), 402; McMillen and Brimnes, ‘Medical Modernisation and Medical Nationalism’. There was also resistance to the work of the ITC in Mexico. See Comstock, *op. cit.*(note 1), 536.10.1590/s1413-8123201100020000421340316

5. L.J. Butler, *Industrialisation and the British Colonial State: West Africa, 1939–51*(London: Frank Cass, 1997), 4.

6. D. George Boyce, *Decolonisation and the British Empire, 1775–1997*(Basingstoke: MacMillan, 1999), 136–8.

7. Ronald Hyam, *Britain’s Declining Empire: The Road to Decolonisation, 1918–80*(Cambridge: Cambridge University Press, 2006), 87. See also Nicholas J. White, *Decolonisation: The British Experience since 1945* (London: Longman, 1999), 7–8.

8. See, for instance, P.J. Cain and A.G. Hopkins, *British Imperialism: Crisis and Deconstruction, 1914–1990*(Harlow: Longman, 1993); Stephen Constantine, The *Making of British Colonial Development Policy, 1914–1940* (London: Frank Cass, 1984); D.J. Morgan, *The Official History of Colonial Development*, 5 vols (Basingstoke: Macmillan, 1980).

9. In addition to the works by Brimnes, the following have examined TB in India and Africa: Sunil Amrith, ‘In Search of a “Magic Bullet” for Tuberculosis: South India and Beyond, 1955–1965’, *Social History of Medicine*, 17, 1 (2004), 113–30; Mark Harrison and Michael Worboys ‘A disease of civilisation: tuberculosis in Britain, Africa and India, 1900–39’, in Lara Marks and Michael Worboys (eds), *Migrants, Minorities and Health: Historical and Contemporary Studies*(London: Routledge, 1997), 93–124; B. Eswara Rao, ‘From Rajayak s(h)ma (Disease of Kings) to Blackman’s Plague: Perceptions on Prevalence and Aetiology of Tuberculosis in Madras Presidency, 1882–1947’, *Indian Economic and Social History Review*, 43, 4 (2006), 457–85; Helen K. Valiers, ‘At home in the colonies: The WHO–MRC trials at the Madras Chemotherapy Centre in the 1950s and 1960s’, in Michael Worboys and Flurin Condrau (eds), *Tuberculosis Then and Now: Perspectives on the History of an infectious Disease* (Ithaca, NY: McGill–Queen’s University Press, 2010), 213–34; Michael Worboys, ‘Tuberculosis and race in Britain and its empire, 1900–50’, in Waltraud Ernst and Bernard Harris (eds), *Race Science and Medicine, 1700–1960* (London: Routledge, 1999), 144–66. On TB in the white dominions, see Alison Bashford, *Imperial Hygiene: A Critical History of Colonialism, Nationalism and Public Health* (Basingstoke: Palgrave Macmillan, 2004), ch. 3; Criena Fitzgerald, *Kissing Can be Dangerous: The Public Health Campaigns to Prevent and Control Tuberculosis in Western Australia, 1900–1960* (Crawley, Australia: University of Western Australia Press, 2006); Maureen K. Lux, ‘Care for the “Racially Careless”: Indian Hospitals in the Canadian West, 1920s–1950s’, *The Canadian Historical Review,* 91, 3 (2010), 407–34.10.3138/chr.91.3.40720857588

10. The first edited collection on the history of Caribbean health and medicine was only published in 2009: Juanita de Barros, Steven Palmer and David Wright (eds), *Health and Medicine in the Circum Caribbean, 1800–1968*(London: Routledge, 2009).

11. See, for instance, Debbie McCollin, ‘World War II to independence: health services and women in Trinidad and Tobago, 1939–1962’, in Barros, Palmer and Wright (eds), *op. cit.*(note 10), 227–48; Darcy Hughes Heuring, *Health and the Politics of Improvement in British Colonial Jamaica*, 1914–1945 (Ann Arbor, MI: Proquest UMI Dissertation publishing, 2012); Margaret Jones, *Public Health in Jamaica, 1850–1940: Neglect, Philanthropy and Development* (Kingston, Jamaica: University of the West Indies Press, 2013).

12. See Jones, *op. cit.*(note 11); McCollin, *op. cit.* (note 11).

13. See, for instance, Sung Lee, ‘WHO and the developing world: the contest for ideology’, in Andrew Cunningham and Bridie Andrews (eds), *Western Medicine as Contested Knowledge*(Manchester: Manchester University Press, 1997), 24–45; Kelley Lee, *The World Health Organization (WHO)* (London: Routledge, 2009).

14. On the work of the Rockefeller in the Anglophone Caribbean, which focuses mainly on the pre-War period, see Steven Palmer, *Launching Global Health: The Caribbean Odyssey of the Rockefeller Foundation*(Ann Arbor, MI: University of Michigan Press, 2010); Rita Pemberton, ‘A Different Intervention: The International Health Commission/Board: Health Sanitation in the British Caribbean, 1914–1930’, *Caribbean Quarterly*, 49, 4 (2003), 87–105; James C. Riley, *Poverty and Life Expectancy: The Jamaica Paradox* (Cambridge: Cambridge University Press, 2005). Existing work on the PAHO has largely focused on the Hispanic Caribbean and Latin America, see Marcos Cueto, *The Value of Health: A History of the Pan American Health Organization* (Washington, DC: Pan American Health Organization, 2007); Anne-Emmanuelle Birn, ‘No more surprising than a broken Pitcher? Maternal and Child Health in the Early Years of the Pan American Sanitary Bureau’, *Canadian Bulletin of Medical History*, 19 (2002), 17–46.10.3138/cbmh.19.1.1711954615

15. The few sources available suggest that in the immediate aftermath of the campaigns the incidence of TB in the region decreased. In Trinidad, for example, there were 610 cases of TB in 1949 declining to 185 in 1958 W.J.Branday, ‘Tuberculosis in Trinidad’, Caribbean Medical Journal, 22, 1–4 (1960), 44.

16. W.Santon Gilmour, Tuberculosis in the West Indies: Report on Sociological and Clinical Survey (London: National Association for the Prevention of Tuberculosis), 26–27.

17. In most colonies, whites made up less than 5% of the total population.

18. Based on Gilmour, *op. cit.*(note 16). On overcrowding in the interwar Caribbean, see Mary Chamberlain, *Empire and Nation-building in the Caribbean: Barbados, 1937–66* (Manchester: Manchester University Press, 2010), 56; Juanita de Barros, *Order and Place in a Colonial City: Patterns of Struggle and Resistance in Georgetown, British Guiana, 1889–1924* (Montreal: McGill-Queen’s University Press, 2002); R. Harris, ‘From Miser to Spendthrift: Public Housing and the Vulnerability of Colonialism in Barbados, 1935 to 1965’, *Journal of Urban History*, 33, 3 (2008), 443–66.

19. Annual Medical Report St Kitts 1954, Table 14; http://www.hpa.org.uk/Topics/InfectiousDiseases/InfectionsAZ/Tuberculosis/TBUKSurveillanceData/TuberculosisMortality/TBMortality01trend/ (accessed 10 June 2013).

20. British Dependencies in the Caribbean and North Atlantic, 1939–1952, 1951–52, Cmd. 8575, xxiv, appendix 2.

21. In Jamaica, for example, the population grew by 2% per year between 1940 and 1950 but employment increased by only 1.3% and some 17.5% of the labour force was unemployed. Wages were about a one third of the UK. Owen Jefferson, *The Post-War Economic Development of Jamaica*(Jamaica: Institute of Social and Economic Research, 1972), 29–30. While some people lacked sufficient food, others had enough to eat but because of their income their diets were unbalanced, lacking animal protein especially, and this made them susceptible to TB. See Chamberlain, *op. cit.* (note 18), 54–5; Riley, *op. cit.* (note 14), 138.

22. G.W.Roberts, The Population of Jamaica (Cambridge: Cambridge University Press, 1957), 161.

23. Gilmour, *op. cit.*(note 16), 135.

24. Cyprus, for instance, only opened its first TB clinic in 1937.

25. Based on annual medical reports; Tuberculosis in the Colonies, Colonial Office (CO) 859/210/5; W.J. Branday, ‘Tuberculosis in Jamaica’, *Caribbean Medical Journal*, 6, 3 (1944), 153–69 and ‘Tuberculosis in Trinidad’, *Caribbean Medical Journal*, 13, 3–4 (1951), 110–27.

26. Until the CDA was passed in 1929, all government expenditure had to be financed by taxpayers’ money or loans. As the CDA aimed to stimulate employment in the UK by providing orders for goods and materials, the public health schemes it funded were capital projects.

27. Michael Havinden and David Meredith, *Colonialism and Development: Britain and Its Tropical Colonies, 1850–1960*(London: Routledge, 1993), 166.

28. On the labour riots, see Thomas C. Holt, *The Problem of Freedom: Race, Labor, and Politics in Jamaica and Britain, 1832–1938*(Baltimore, MD: Johns Hopkins University Press, 1992); Nigel O. Bolland, *On the March* (Kingston, Jamaica: Ian Randle Publishers, 1995).

29. Until the riots, all Caribbean colonies with the exception of Barbados were Crown colonies. The Legislative Council in some was made up of appointed members and a few elected on the basis of a narrow franchise but in others it consisted solely of appointed (local and ex-pat) members. The Executive Councils in all Crown colonies were fully appointed.

30. The CDWA widened the scope of development projects in the Empire because in contrast to the CDA it included operating costs as well as capital expenditure. See E.R. Wicker, ‘Colonial Development and Welfare, 1929–1957: The Evolution of a Policy’, *Social and Economic Studies*, 7, 4 (1958), 170–92; Howard Johnson, ‘The West Indies and the Conversion of the British Official Class to the Development Idea’, *Journal of Commonwealth and Comparative Politics*, 15 (1977), 55–83.

31. Based on annual medical reports; Branday, ‘Tuberculosis in Trinidad’ (1951), *op. cit.*(note 25) and ‘Tuberculosis in Trinidad’ (1960), *op. cit.* (note 15), 41–4; Richard A.S. Cory, ‘Special Treatment of Respiratory Tuberculosis over a Quarter of a Century in Jamaica, West Indies’, *Caribbean Medical Journal*, 22, 1–4 (1960), 75–80; H.P. Fernandes, ‘Streptomycin in the Treatment of Tuberculosis in British Guiana’, *Caribbean Medical Journal* 13, 3–4 (1951), 51–78.

32. Branday, ‘Tuberculosis in Trinidad’ (1951), *op. cit.*(note 25) and ‘Tuberculosis in Jamaica’ *op. cit.* (note 25); T.R. Jones, ‘Tuberculosis in British Guiana’, *Caribbean Medical Journal*, 22, 1–4 (1960), 105–11.

33. Gilmour, *op. cit.*(note 16), 133–9 and 157–66; Annual Medical Report Grenada 1954, 3–4; Annual Medical Report St Kitts 1954, 10–11.

34. In many colonies, it was not until the late 1930s or even after the CDWA was passed that TB services were developed. TB work in Fiji, for instance, only started in 1943 with a survey funded by the CDWA, followed three years later by the establishment of a sanatorium. In African colonies especially, TB services were far less developed than in the Caribbean. For example, in the Gold Coast, where the disease was particularly rife in the mining areas, none of the hospitals had a TB ward in 1947 and the colony also lacked a mobile X-ray unit. See ‘Tuberculosis in the Colonies’, CO 859/210/5; NAPT, *Tuberculosis in the Commonwealth 1947*(London: NAPT, 1947), 249–53.

35. Annual Medical Report St Kitts 1954, 11. The TB schemes in the big three, then, largely mirrored the vertical, specialist programme in place in the UK at the time. See Linda Bryder, *Below the Magic Mountain: A Social History of Tuberculosis in Twentieth Century Britain*(Oxford: Clarendon Press, 1988).

36. Jamaica, for instance, spent £30,000 on TB work in 1944, most of which went towards salaries. Branday, ‘Tuberculosis in Jamaica’, *op. cit.*(note 25), 168.

37. Colonial Development and Welfare Acts,1949–50, House of Commons Papers (107), xix; 286–96; Colonial Development and Welfare Acts, 1950–51, House of Commons Papers (189), xxvi, Colonial Development and Welfare Acts, 1951–52, House of Commons Papers (211), xxiv; Colonial Development and Welfare Acts, 1952–53, House of Commons Papers (189), xxiii; Colonial Development and Welfare Acts, 1953–54, House of Commons Papers (181), xxv. On the shortcomings of the CDWA-funded programmes, see Chamberlain, *op. cit.*(note 18), ch. 3.

38. *The Gleaner*, 22 May 1948 and 5 November 1949; I.J. Rerrie, ‘Review of Adult Tuberculosis in Jamaica’, *Caribbean Medical Journal*, 22, 1–4 (1960), 66; Annual Medical Reports Jamaica 1948–1950.14491535

39. Administration Report of the Public Department of the City of Port of Spain 1948, 16; Branday, ‘Tuberculosis in Trinidad’ (1951), *op. cit.*(note 25), 121; E. Losonczi, ‘Report on the BCG campaigns in British Honduras’, *West Indian Medical Journal*, 5, 4 (1956), 271; WHO/TBC/Int./32 (St Kitts), 10.13423228

40. Linda Bryder, “‘We shall not find Salvation in Inoculation”: BCG Vaccination in Scandinavia, Britain and the USA, 1921–1960’, *Social Science and Medicine*, 49 (1999), 1163; Branday, ‘Tuberculosis in Jamaica’, *op. cit.*(note 25), 166.10.1016/s0277-9536(99)00157-410501638

41. British Dependencies in the Caribbean and North Atlantic, 56–64.

42. The Colonial Territories, 1949–50, Cmd. 7958, viii, 113. Various TB surveys had been carried out during the War with support from the CDWA and NAPT, including those in the Caribbean, Cyprus, Fiji and Tanganyika (Kenya).

43. NAPT, *Tuberculosis in the Commonwealth*, *op. cit.*(note 25), 146. See also the lectures by Dr Esmond Ray Long, Dr Arthur Quinton Wells and Dr Kenneth Neville Irvine in the same volume.

44. ‘Commonwealth and Empire Tuberculosis Conference’, *British Medical Journal*, 2, 4619 (1949), 154.

45. Before the Second World War, public health authorities in the UK had been very sceptical about BCG. They not only questioned both the safety and efficacy of the vaccine but also feared that inoculation would interfere with the country’s well-developed and fairly successful TB control programme. It was largely a shortage of nurses for TB institutions that led TB experts in 1943 to ask the Ministry of Health to both supply and study BCG. Reports of the trial with schoolchildren were published in the *British Medical Journal* in 1959 and 1963 and concluded that BCG offered substantial protection. Bryder, ‘We shall not find salvation in inoculation’, 1161–62.

46. In 1950, Heaf paid several visits to the Pasteur institute in Paris to learn more about freeze-dried BCG, which was seen as less effective than the live vaccine but was not as widely studied.

47. See, for instance, the demands from Northern Rhodesia, Ceylon (Sri Lanka) and Singapore in CO 859/210/6 and CO 859/216/1.

48. Dr R. Gautier to Dr Melville MacKenzie, 13 September 1948, CO 859/216/1; Dr Wilson Rae to Dr Melville MacKenzie, 13 September 1948, CO 859/216/1. By 1948, Jamaica and Trinidad had adopted universal suffrage and in Barbados and Guyana the franchise had been considerably extended. In addition, both Jamaica and Barbados had a quasi-ministerial system and in Trinidad and Guyana the number of elected members on the Legislative Council was increased and they were also allowed to sit on the Executive Council. Less constitutional progress was made in St Kitts and Grenada but even here the voice of the local members of the Legislative Council was taken more into account. Morley Ayearst, *The British West Indies: The Search for Self-Government*(London: Allen and Unwin, 1960), chs 4–5.

49. World Health Organization suggestions for the control of tuberculosis in countries with undeveloped or underdeveloped programmes, CO 859/210/5. On the Colonial Office’s responses to this plan, see Extract from CAMC minute of 453rd meeting held 28.3.50, CO 859/210/5. On WHO’s adoption of BCG, see Brimnes, ‘BCG’, *op. cit.*(note 2), 865–7.

50. Colonial Territories, 1949–50, 113–14; Colonial Territories 1950–51, Cmd. 8243, xxvi, 134; Professor Heaf to Dr Priddie, 2 October 1950, CO 859/201/6; Notes on BCG vaccine by Dr Rae, 6 February 1950, CO 859/201/6.

51. On the Cold War in the British Caribbean, see Gerald Horne, *Cold War in a Hot Zone: Labor and Independence Struggles in the British West Indies*(Philadelphia, PA: Temple University Press, 2007); Spencer Mawby, *Ordering Independence: The End of Empire in the Anglophone Caribbean, 1947–49* (Basingstoke: Palgrave Macmillan, 2012).

52. Telegram to the Secretary of State for the Colonies from the Acting Governor, 22 September 1950, CO 936/55/1; Message from his Excellency the Acting Governor to the honourable House of Representatives, 10 October 1950, CO 936/55/1.

53. Teams were made up largely of local people, some of whom had been given a travelling scholarship to observe field work elsewhere. Before they started work in a new area, teams first undertook a publicity campaign and also met with local authorities. For more on the intensive method, see Palmer, *op. cit.*(note 14), ch. 4; Pemberton, *op. cit.* (note 14), 91–3.

54. Wilson to Washburn, 14 July 1927 and Wilson to Howard, 5 December 1927, Rockefeller Foundation (RF), RG 5, series 1.2, box 302, folder 3831.

55. *The Gleaner*, 10 May 1934.

56. *The Gleaner*, 12 October 1950; Telegram to the Secretary of State for the Colonies from the Acting Governor, 22 September 1950, CO 936/55/1.

57. Inward telegram to the Secretary of State for the Colonies from the Acting Governor, 22 September 1950, CO 936/55/1; Message from his Excellency the Acting Governor to the honourable House of Representatives, CO 936/55/1.

58. Acting Governor to UK delegations at the United Nations, 20 October 1950, CO 936/55/1.

59. Hyam, *op. cit.*(note 7), 178.

60. Note by executive director on the request of Jamaica for UNICEF assistance in a BCG programme, 1 November 1950, CO 936/55/1. Plans for the ITC take-over by UNICEF/WHO were discussed at the 5th session of WHO’s expert committee on tuberculosis in September 1950.

61. Savingram from New York to Foreign Office, 2 December 1950, CO 936/55/1.

62. Recommendation of executive director for an appointment to Jamaica, CO 137/899/3; Savingram from New York to Foreign Office, 2 December 1950, CO 936/55/1.

63. Acting Governor to Secretary of State for the Colonies, 27 February 1951, CO 137/899/3.

64. Acting Governor to UK delegations at the United Nations, 20 October 1950, CO 936/55/1; Tuberculosis (prevention, diagnosis and ambulatory treatment) scheme, CO 936/55/1.

65. *Ibid*.

66. Telegram from the Secretary of State for the Colonies to the Governor of Jamaica, 19 June 1951 CO 137/899/3; Comptroller Development and Welfare Organisation to the Secretary of State for the Colonies, 16 November 1950, CO 137/899/3; Professor Heaf to Dr Rae, 15 March 1951, CO 137/899/3; Memorandum by DMS on comments by Professor Heaf, 7 April 1951, CO 137/899/3; Dr L.W. Fitzmaurice to Dr Fred L. Soper, 3 February 1951, CO 137/899/3. Jamaica’s national income had grown from £39.9 million in 1943 to £85 million in 1950. The 1940 CDWA gave the Secretary of State for the Colonies authority to make schemes likely to promote the welfare of the people in the colonies but in ‘concurrence with the Treasury’. Hence, many CDWA proposals were, like the Jamaican demand for support for a BCG vaccination campaign. Wicker, (note 30), 186.

67. See, for instance, Telegram from New York to Foreign Office, 6 June 1951, CO 137/899/3; Governor of Jamaica to Colonial Office representative with the permanent United Kingdom delegation to the United Nations, 1 June 1951, CO 137/899/3; E.C. Burr to Miss A.E. Murray, 13 June 1951, Foreign Office (FO) 371/95900; Confidential letter Foreign Office to United Kingdom Delegation to the United Nations, 30 June 1951, CO 137/899/3; Foreign Office to United Kingdom Delegation to the United Nations, 24 July 1951, CO 137/899/3; United Nations (economic and social) department to UK delegation to the United Nations, 14 September 1951, CO 137/899/3; Agreement between the United Nations International Children’s Emergency Fund and His Majesty’s Government in the United Kingdom of Great Britain and Northern Ireland with respect to Jamaica, 4 October 1951 CO 137/899/3.

68. The UK differed in this regard from France, which was more reluctant to grant UN agencies access to its colonies. See, Hyam, *op. cit.*(note 7), 180–1; John Kent, *The Internationalization of Colonialism: Britain, France, and Black Africa, 1939–1956* (Oxford: Clarendon Press, 1992), 267–9.

69. Losonczi, *op. cit.*(note 39), 271.

70. Savingram from the Secretary of State for the Colonies to the Governor of Trinidad, 20 July 1951, FO 371/95900.

71. In 1952, a TB survey was undertaken in Barbados. It was decided, however, to make a BCG campaign part of a wider package of UNICEF/WHO support, including a mass VD campaign, an agreement for which was signed in 1954 and implemented from 1955 onwards. WHO (Project files), Barbados 2, 2109.A. 5021/8.1/54.

72. Brimnes, ‘Vikings against Tuberculosis’, *op. cit.*(note 2), 420–9.

73. The 1950 campaign in Syria, for example, employed four local and six international medical staff.

74. Annual Medical Report Jamaica 1951. There were also many private doctors in the British Caribbean at the time.

75. ‘BCG Vaccination on a World-Wide Scale’, *Public Health Reports*, 66, 36 (1951), 1160.14864810

76. Based on the reports of the campaigns (hereafter WHO reports) at the WHO archives in Geneva: WHO/TBC/Int./33 (Honduras); WHO/TBC/Int./31 (Grenada); WHO/TBC/Int./37 (Jamaica); WHO/TBC/Int./32 (St Kitts); WHO/TBC/Int./34 (Trinidad). WHO archives do not contain the reports of the Barbados and Guyanese campaigns. Information about these campaigns is derived from: Annual Medical Reports Barbados 1955–56 and 1956–57 and Annual Medical Report Guyana 1955.

77. See WHO reports and *The Gleaner*, 13 September 1955.

78. West India Royal Commission Report, 1944–45, Cmd 6607, vi, 157–8.

79. *Ibid*., 158.

80. See *The Gleaner*, 18 August 1955, 3 July 1961 and 8 March 1962.

81. On the standardised procedure developed for the campaign see, *Final Report of the International Tuberculosis Campaign, July 1, 1948 to June 30, 1951*(Copenhagen: International Tuberculosis Campaign, 1951), Part 1.

82. Based on WHO reports.

83. *Ibid*.

84. Losonczi, *op. cit.*(note 39), 271.

85. WHO report Jamaica, 2; WHO report St Kitts, 2.

86. WHO report Jamaica, 4.

87. *Ibid.*, 4; WHO report St Kitts, 3.

88. WHO reports and Annual Medical Report Barbados, 1956–57, 11; Annual Medical Report Guyana 1955, 8. The variation can largely be explained by a colony’s degree of urbanisation – urban areas had a higher coverage rate than rural areas – and infrastructure.

89. ‘Statistical report of UNICEF/WHO BCG Vaccination Programmes’, *Bulletin World Health Organization*, 12 (1955), 307–8.PMC254232014351981

90. Comstock, *op. cit.*(note 1), 535.

91. British Dependencies in the Caribbean and North Atlantic, 53.

92. *The Gleaner*, 14, 15 and 21 March 1952.

93. Statistical report of UNICEF/WHO BCG Vaccination Programmes’, 305; Comstock, *op. cit.*(note 1), 536.

94. Annual Medical Report Jamaica 1952, 5.

95. Annual Medical Reports Guyana 1955 and 1956.

96. Annual Medical Report Honduras 1953.

97. See, for instance, Annual Medical Report Barbados 1956–57.

98. In its first decade, WHO was concerned with the prevention of communicable diseases and devoted much energy to scientific research and promotion of medical education. See Brimnes, ‘BCG’, *op. cit.*(note 2), 866; R.M. Packard, “‘No other Logical Choice”: Global Malaria Eradication and the Politics of International Health in the Post-War Era’, *Parassitologia*, 40 (1998), 218; Javed Siddiqi, *World Health and World Politics: The World Health Organization and the UN System* (Columbus, SC: University of South Carolina Press, 1995), 193–7; Amy L.S. Staples, *The Birth of Development: How the World Bank, Food and Agriculture Organization, and World Health Organisation Changed the World, 1945–1965* (Kent, Ohio: Kent State University Press, 2006), 138–9; WHO, *The First Ten Year of the World Health Organization* (Geneva: WHO, 1958).

99. WHO report Honduras, 5; Annual Medical Report Honduras 1953, 6.

100. Annual Medical Report Barbados 1957–58, 36; Annual Medical Report Barbados, 1960–61, 36. Follow up campaigns were also carried out in Guyana and Grenada.

101. Branday, *op. cit.*(note 15), 46; Annual Medical Reports Guyana 1955 to 1958 and 1960 and 1961.

102. Rerrie, *op. cit.*(note 38), 68.

103. *Ibid*; Rerrie, ‘Childhood Type Tuberculosis in Jamaica, *Caribbean Medical Journal*, 22, 1–4 (1960), 73.14491536

104. Branday, *op. cit.*(note 15), 46. The Guyanese BCG team visited 171 schools in 1961 but also failed to visit all schools on an annual basis. Annual Medical Report Guyana 1961, 13.

105. British Dependencies in the Caribbean and North Atlantic, appendices 4–14.

106. See, for instance, Dorothy Porter, ‘How soon is now? Public Health and the *BMJ*’, *BMJ*, 301, 6754 (1990), 738–40.10.1136/bmj.301.6754.738PMC16640652224250

107. West India Royal Commission report, 1944–45, Cmd 6607, vi, 141–2.

108. For more on class and racial biases in the provision of health care in the colonial Caribbean, see, Henrice Altink, ‘Modernity, Race and Mental Health Care in Jamaica, *c*1918–1944’, *Journal of the Department of Behavioural Sciences*, 2, 1 (2012) at http://journals.sta.uwi.edu/jbs/index.asp?action=viewAbstract&articleId=312 (accessed at 10 June 2013); Margaret Jones, ‘A “Textbook Pattern”? Malaria Control and Eradication in Jamaica, 1910–65’, *Medical History*, 57, 3 (2013), 397–419.10.1017/mdh.2013.20PMC386594624069886

109. See for instance, Mission to Jamaica (International Bank for Reconstruction and Development), *The Economic Development of Jamaica*(Baltimore, MD: Johns Hopkins Press, 1952), 121. On the black middle-class’s prejudices against the lower and invariably darker-skinned classes, see Eric Williams, *The Negro in the Caribbean* (New York: Haskell House Publishers, 1942; Westport, CT: Greenwood Press, 1969), 61.

110. Amrith, *op. cit.*(note 9).

